# Visual processing speed and its association with future dementia development in a population-based prospective cohort: EPIC-Norfolk

**DOI:** 10.1038/s41598-024-55637-x

**Published:** 2024-02-29

**Authors:** Ahmet Begde, Thomas Wilcockson, Carol Brayne, Eef Hogervorst

**Affiliations:** 1 School of Sport, Exercise and Health Sciences, Loughborough University, Loughborough, LE11 3TU UK; 2 Department of Public Health, University of Cambridge, Cambridge, Cambridgeshire CB2 1PZ UK

**Keywords:** Dementia, Alzheimer's disease

## Abstract

Visual processing deficits have frequently been reported when studied in individuals with dementia, which suggests their potential utility in supporting dementia screening. The study uses EPIC-Norfolk Prospective Population Cohort Study data (n = 8623) to investigate the role of visual processing speed assessed by the Visual Sensitivity Test (VST) in identifying the risk of future dementia using Cox regression analyses. Individuals with lower scores on the simple and complex VST had a higher probability of a future dementia diagnosis HR1.39 (95% CI 1.12, 1.67, P < 0.01) and HR 1.56 (95% CI 1.27, 1.90, P < 0.01), respectively. Although other more commonly used cognitive dementia screening tests were better predictors of future dementia risk (HR 3.45 for HVLT and HR 2.66, for SF-EMSE), the complex VST showed greater sensitivity to variables frequently associated with dementia risk. Reduced complex visual processing speed is significantly associated with a high likelihood of a future dementia diagnosis and risk/protective factors in this cohort. Combining visual processing tests with other neuropsychological tests could improve the identification of future dementia risk.

## Introduction

For many decades there has been interest in whether there are any reliable markers that can be measured which predict sufficiently accurately future dementia to be considered with singly or together with other measuers. Dementia itself is characterised by impairment in memory and other cognitive functions impacting on activities of daily life^1,2^. Some researchers suggest that early diagnosis of dementia would play a critical role in identifying people at risk of complications and preventing the progression of the disease by planning and implementing early treatment strategies^3^. Others, however, recommend timely diagnosis only until the effects of early diagnosis and support are more thoroughly investigated to assess benefits and harms^4^. While brain scans, psychiatric evaluations, genetic and blood tests for biomarkers might be used to support a diagnosis of dementia^5^, the syndrome remains a clinical diagnosis and an important part of this is established and objective cognitive and neuropsychological tests, such as Mini Mental State Examination (MMSE), Montreal Cognitive Assessment (MoCA) and Hopkins Verbal Learning Test (HVLT). In Western societies, these types of tests are recommended as crucial for the screening of dementia even in the early stages^6,7^. In addition to these tests for global cognitive and memory impairment, it could be worth considering the assessment of visual processing ability for early screening of dementia risk, as visual processing ability is often negatively impacted in the early stages^8,9^.

Visual deficits in people with dementia have been found in both lower-level visual processing, such as contrast sensitivity, colour vision, simple perception, and visual acuity^10^, and higher-level visual processing, including visual integration and visuospatial functions in experimental studies^11^. Previous observational studies have also reported an association between visual impairment and the increased risk of future dementia^12,13^. In addition, data from two large cohort studies reported that impairment in visual acuity was associated with a dose dependent increased risk of dementia, seen in people with mild (HR 1.26, 95% confidence interval (CI) 0.92–1.72) to severe visual impairment (HR 2.16, 95% CI 1.37–3.40)^14^. Similarly, another cohort study found the greatest risk (among other risk factors) for dementia (HR  2.14–5.66) and mild cognitive impairment (HR  6.43, 95% CI 1.66–24.85) among people with poor visual acuity^15^. Therefore, the association between dementia and broad range of visual deficits seems apparent and warrants further investigation.

Some neuropsychological tests, such as the Addenbrookes Cognitive Examination and the MoCA, include elements of perceptual processing and visuospatial functions^16,17^. However, these screening tests are not comprehensive enough to enable the assessment of different functions, such as visual processing speed, hand–eye coordination, contrast sensitivity and visuo-constructional ability. Therefore, visual sensitivity tests that contain different visual processing components and assess visual deficits contributing to cognitive impairment might be an effective way to identify risk for early dementia.

The aim of the study is to validate the Visual Sensitivity Test (VST) against two gold-standard dementia screening tools: the HVLT and Short Form Extended Mental State Exam (SF-EMSE). The VST is a standardized assessment tool utilized for evaluating visual processing speed and simple and complex motor response time in individuals^18^. This test evaluates not only basic processing speed, but also more intricate processes, such as visual contrast sensitivity and complex visuospatial perceptual ability among moving distractors. Previous research findings by Hayat et al.^19^ showed that VST scores of individuals at risk for future dementia were significantly lower when compared to cognitively intact individuals, indicating an association between poor VST performance and early dementia risk. The present study aims to assess whether the VST using clinical cut-off scores has good sensitivity and specificity for the risk of future dementia and whether performance below the cut-off score is associated with patient characteristics, co-morbidities, self-reported health status and physical activity levels^20^ or is independently related to dementia risk.

## Methods

The present study used data from the European Prospective Investigation into Cancer Norfolk study, a population-based prospective cohort study (The EPIC-Norfolk Study, 2021). The study recruited 25,639 people (aged 40–79) living in the county of Norfolk, United Kingdom, an area that includes a small city, market towns and a rural population between 1993 and 1997. Afterwards, follow-up assessments were held at regular intervals in which the health and behaviours of the participants were measured. In the third health check (3HC), cognition, physical functioning, and eye health of 8623 participants aged 48–92 years were assessed between 2004 and 2011. The data collected in the 3HC is used in this present study^21^. The study was approved by the Norwich District Ethics Committee (05/Q0101/191) and East Norfolk and Waveney NHS Research Governance Committee (2005EC07L). Informed consent was obtained from all participants. All experiments were performed in accordance with relevant guidelines and regulations.

Several cognitive tests were used in the EPIC-Norfolk 3HC; the VST with two parts (simple and complex VST), the HVLT and the SF-EMSE. The tests have been described in detail previously^21^. Briefly, the VST assesses visual processing speed and reaction time^18^. Participants were instructed to hit the space bar as soon as they detected a triangle (simple VST). Second version of the test requires responding to a triangle (pointing upside or downside) formed from constantly moving dots anywhere and randomly in the visual field (complex VST). The reaction time (RT in msec) was recorded. The HVLT measures immediate recall and verbal learning using a 12-item word list of three semantic categories presented 3 times to provide a learning curve^7,22,23^. The participants were instructed to listen carefully as the assessor read the word list and to try to recall the words in any order. The participants’ free recall of the list was recorded. The same procedure was performed twice more. After the third learning attempt, the total number of immediately recalled words was noted. The SF-EMSE is a 26-item test to assess global cognition and functioning at the higher end of the ability range^21^. Dementia was defined during a follow-up period from 2006 to 2019 using hospital inpatient records received from Hospital Episode Statistics, death certificates and mental health data. The data were coded using the International Classification of Diseases version 10^24^.

Age (in years), gender, marital status, education level and residential area deprivation as an indicator of socioeconomic status (SES) were included as socio-demographic factors known to be associated with dementia risk^25^. Marital status was categorised as either (1) married (married) or (2) single (single, widowed, separated or divorced). Education was categorised into three groups based on the highest qualification attained: (1) no qualification, (2) ordinary or advanced level (O-A level) or (3) graduate level. The Townsend deprivation index was used to define residential area deprivation. The Index was divided into five equal quintiles. A lower Townsend score signifies a lower level of deprivation, with quintiles 1–4 being below the average for England and Wales. Quintile 5, which ranges from − 0.64 to 6.99, represents a level of deprivation close to or above the national average.

Self-reported physical activity level was measured with a single question asking about the amount of time spent on physical activity (in hours/week). Self-rated health status was assessed using the 36-item short-form questionnaire (the SF-36). The total score for this ranged from 0 (poor health) to 100 (good health)^26^. Co-morbidities (cancer, stroke, heart attack, diabetes, depression, visual problem, hearing problem) were established using a self-report questionnaire (with 0 = no and 1 = yes, present).

### Analyses

The characteristics of groups, dementia and no dementia at follow-up, were compared using descriptive statistics (using Chi-square tests for percentages, Mann Whitney-U tests for not normally distributed variables and independent t-tests for normally distributed variables) (see Table 1). A significance level of P < 0.05 (2-tailed) was used to determine statistical significance.

**Table 1:** Characteristics of 8585 participants with cognitive measures in the third health check phase of the European Prospective Investigation of Cancer in Norfolk (EPIC-Norfolk) study, 2006–2011 (including Pilot Data, 2004–2006), followed up until 31 March 2019, stratified by dementia status.

Variables	All participants (n = 8585)	Dementia (n = 537)	No dementia (n = 8048)	*P* value
Age (years), mean (SD)	68.7 (8.1)	76.3 (6.2)	68.2 (7.9)	P < 0.01**
Gender, n (%)				P = 0.02*
Women	4747 (55%)	271 (51%)	4476 (56%)	
Men	3838 (45%)	266 (49%)	3572 (44%)	
Marital status, n (%)				P < 0.01**
Married	6556 (78%)	156 (30%)	1666 (21%)	
Single	1822 (22%)	358 (70%)	6198 (79%)	
Education level, n (%)				P < 0.01**
No qualifications	2249 (26%)	184 (34%)	2065 (25%)	
O-A level	4815 (56%)	274 (51%)	4541 (57%)	
Graduate level	1513 (18%)	79 (15%)	1434 (18%)	
Townsend Deprivation Index, mean (SD)	-2.7 (2.1)	-2.1 (2.2)	-2.3 (2.1)	P = 0.04*
Physically Activity Level, self-report (hrs/wk), mean (SD)	26 (14.9)	25 (15.9)	26.1 (14.9)	P = 0.04*
SF36 Physical Functioning, mean (SD)	77.5 (22.7)	68.7 (25.3)	78.1 (22.4)	P < 0.01**
Comorbidity, self-report, n (%)				
Cancer	659 (9%)	41 (9%)	618 (9%)	P = 0.09
Stroke	105 (1%)	13 (3%)	92 (1%)	P < 0.01**
Heart attack	225 (3%)	15 (3%)	210 (3%)	P = 0.73
Diabetes	229 (3%)	31 (7%)	198 (3%)	P < 0.01**
Depression	1207 (16%)	66 (14%)	1141 (16%)	P = 0.23
Visual problem	2103 (29%)	173 (42%)	1930 (29%)	P < 0.01**
Hearing problem	2714 (32%)	221 (43%)	2493 (32%)	P < 0.01**
Cognitive functions, mean (SD)				
HVLT	25.1 (5.7)	19.9 (6.4)	25.4 (5.5)	P < 0.01**
VST-simple	663 (166)	722 (224)	660 (161)	P < 0.01**
VST-complex	2197 (429)	2379 (501)	2185 (421)	P < 0.01**
EMSE	35.6 (3.11)	29.8 (4.5)	32.8 (2.9)	P < 0.01**

Significant values are in [bold].

*P < 0.05, **P < 0.01.

Receiver operating characteristics (ROC) curves were produced to examine the discriminative validity for detecting future dementia and compared the VST with the commonly used assessments for dementia, the HVLT and SF-EMSE. The Youden's index was used to determine the optimal cut-off scores for the highest sensitivity and specificity obtained with the ROC analyses. The risk of future dementia was assessed by calculating the hazard ratio (HR) with a 95% confidence interval (95% CI) separately for VST, HVLT, and SF-EMSE using Cox proportional hazard regression models. These models were adjusted for socio-demographic factors, specifically age, sex, and education.

Spearman’s rank correlations were performed to identify clusters of associations followed by logistic regression analyses using the most optimal cut-off scores of the VST-complex as the dependent variable. After entering dementia diagnosis, potential confounds significantly correlated with the dependent variable (age, gender, education etc.) were included as independent variables. Similar ROC and logistic regression analyses were done for the HVLT and SF-EMSE which were previously found to distinguish well between dementia cases and controls^6,7^. The exclusion criterion for variables was a significance level of P > 0.05. The Nagelkerke R^2^ value and the Hosmer and Lemeshow goodness-of-fit test were used to evaluate the derived models. The results of both VSTs were subjected to a log transformation to better align with Gaussian distributions^27^. All statistical analyses were performed using IBM SPSS 27 statistical software.

## Results

Of the 8623 participants included in 3HC, 8585 men and women aged 48–92 at the time of their cognitive assessment completed at least one of the 3 cognitive tests from 2004 to 2012. Based on ICD codes, 533 participants were diagnosed with dementia at follow-up period of up to 14.8 years. The average time between the date of cognitive testing and the date of diagnosis is of 9.6 years (from 2004 to 2019), with a median of 9.8 years. The missing data rate of the variables in the dataset ranged from 0 to 18%. Table 1 compares the characteristics of people with dementia and people who were not diagnosed with dementia at follow-up. People living with dementia were likely to be in an older age group, were less educated, less physically active, and had worse self-reported general health status. As expected, self-reported co-morbidities, such as diabetes, stroke, visual and hearing problems at baseline, which are important risk factors for dementia, were also more common in people with dementia. Likewise, on all 3 cognitive tests, people in the dementia group had significantly worse scores than the no-dementia group.

Cox regression analyses were used to analyse the association between reduced visual processing speed at baseline and future dementia development. Low visual processing speeds (both simple and complex) were associated with an increased risk of future dementia (HR 1.39, 95% CI 1.12–1.67, P < 0.01 and HR 1.56, 95% CI 1.27–1.90, P < 0.01; respectively, see Table 2). Poor scores in HVLT (HR 3.45, 95% CI 2.85–4.17, P < 0.01) and SF-EMSE (HR 2.66, 95% CI 2.23–3.18, P < 0.01) were also significant predictors of future dementia (Table 2). Sensitivity analyses using alternative cut-offs (10th percentile) showed that reduced VST scores remained significant predictors of future dementia, with an increase in hazard ratios (from 1.39 to 1.77 for the simple VST and from 1.56 to 2.15 for the complex VST).

**Table 2:** Cox regressions for the association of cognitive measures and the risk of future dementia.

Cognitive tests, (n) (ref. = low score)	Dementia (n)	Hazard ratio (95% CI)	*P* value
VST-simple (7169)	413	1.39 (1.12, 1.67)	P < 0.01**
VST-complex (7169)	413	1.56 (1.27, 1.90)	P < 0.01**
HVLT (8135)	485	3.45 (2.85, 4.17)	P < 0.01**
SF-EMSE (8479)	521	2.66 (2.23, 3.18)	P < 0.01**

Significant values are in [bold].

Ref: lower score of cut-off point was used as the reference group for comparison. All models adjusted for age, sex, and education.

Subgroup analyses were performed to examine the association between visual processing speed and future dementia risk while accounting for age differences (see supplementary material, Appendix A). Participants younger than 75 years were excluded, and the analyses were conducted on the remaining subset aged 75 and older. Of the original 8585 participants, 2038 were included in these subgroup analyses, among which 322 developed dementia during the follow-up period. The average age of the 1716 controls was 79.58 years, while those with dementia had a mean age of 80.38 years. After controlling for age in these subgroup analyses, the results remained consistent with the original findings. The HRs for future dementia risk were as follows: VST-simple HR 1.36 (95% CI 1.04–1.77), VST-complex HR 1.47 (95% CI 1.12–1.92), HVLT HR 2.84 (95% CI 2.23–3.61), SF-EMSE HR 2.54 (95% CI 2.01–3.20). These findings indicate that the association between lower VST performance and increased risk of future dementia persists even when accounting for age differences between the groups. The observed deficits in VST performance are not solely driven by age but are indeed associated with an elevated risk of future dementia development.

ROC analyses were performed to compare and illustrate the screening performance of the VST, HVLT and SF-EMSE tests (see supplementary material, Appendix B). The simple and complex VST showed lower area under the curve (AUC) with a value of 0.60 and 0.62, respectively, while the HVLT and SF-EMSE represented slightly higher and similar AUC (0.74 and 0.72, respectively). With the optimal cut-off score of 6.48, the simple VST showed a low sensitivity of 53% and specificity of 63% for future dementia risk. The complex VST had a similar sensitivity of 57% and same specificity of 63% for future dementia risk with an optimal cut-off score of 7.72, whereas the sensitivity and specificity of HVLT (65% and 72%, respectively with a score of 20.5 on the immediate recall as the most optimal cut-off score) and SF-EMSE were slightly higher (68% and 64%, respectively for a score of 32.5 as the optimal cut-off score).

**Figure 1:**
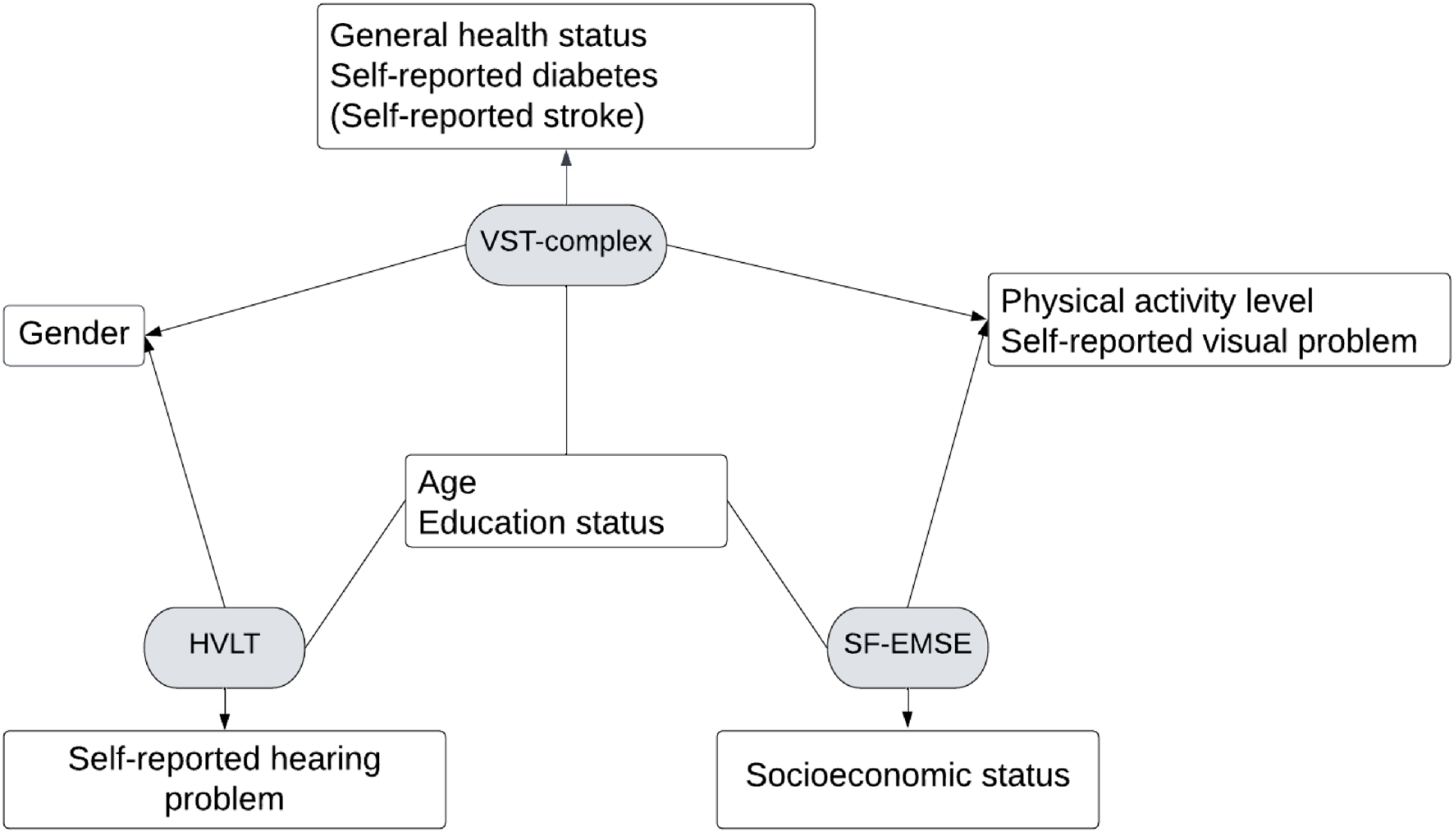
Correlation map showing factors related to cognitive tests.

Using these cut-off scores, categorical variables of two groups (categorised as being below the lower cut-off or above the higher cut-off) were created for each test. Logistic regression results, which included only independent variables that were significantly correlated with the dependent variables (see supplementary material, Appendix C1-Spearman’s correlation matrix), showed that the complex VST was sensitive to variables often implicated in dementia risk studies more than the other two tests (see Table 3). Although the simple VST only captured age, gender, and education as significant factors (see Table 4), the complex VST lower scores were independently predicted by variables including an older age, the female gender, lower education status, worse general health status, self-reported diabetes, lower physical activity levels and self-reported visual problems, which are important and are considered to be dementia risk factors based on previous studies^25^ (see Fig. 1). For the HVLT cut-off low scores were associated with an older age, being a woman, lower education and having a self-reported hearing problem (see Fig. 1 and supplementary material, Appendix D1) while the SF-EMSE, an older age, lower SES and education, engaging less in physical activity and self-reported visual problems (Fig. 1; supplementary material, Appendix D1). The results of the logistic regression analyses indicated that the complex VST and HVLT measures were found to be more robust compared to the SF-EMSE measure. When the dementia diagnosis was included as an independent variable in the logistic regression analysis, it was observed that there was no alteration in the predicted variables for the complex VST and HVLT measures. However, the inclusion of the dementia diagnosis resulted in the removal of significant factors, such as physical activity, visual problems, and SES from the model predicting low S-EMSE scores.

**Table 3:** Logistic regression model for VST-complex (CI = 95%).

Variables	OR (95% CI)	*P *value
Age (years)	1.04 (1.03–1.05)	P < 0.01**
Gender (ref = men)	0.80 (0.70–0.90)	P < 0.01**
Marital status^#^ (ref = married)	0.90 (0.80–0.99)	P = 0.72
Education status (ref. = No qualification)	–	P < 0.01**
Education status (O-A level)	0.84 (0.73–0.97)	P < 0.01**
Education status (graduate)	0.74 (0.62–0.89)	P < 0.01**
Townsend DI^#^	1.05 (0.99–1.11)	P = 0.32
Self-reported stroke (ref = no)	0.69 (0.40–1.20)	P = 0.06
Self-reported diabetes (ref = no)	0.63 (0.45–0.90)	P < 0.01**
Physical activity (ref = active)	0.97 (0.95–0.99)	P = 0.02*
General health status^#^ (ref = poor)	1.12 (0.94–1.34)	P = 0.20
Visual problem (ref = yes)	1.20 (1.06–1.38)	P < 0.01**
Hearing problem^#^ (ref = yes)	1.00 (0.88–1.14)	P = 0.96

Significant values are in [bold].

^#^Variable removed from the model, *P < 0.05, **P < 0.01.

**Table 4:** Logistic regression model for VST-simple (CI = 95%).

Variables	OR (95% CI)	*P *value
Age (years)	1.05 (1.04–1.06)	P < 0.01**
Gender (ref = men)	0.90 (0.80–1.01)	**P < 0.04**
Marital status^#^ (ref = married)	0.99 (0.85–1.15)	P = 0.90
Education status (ref. = No qualification)	–	P < 0.01**
Education status (O-A level)	0.86 (0.73–0.99)	P < 0.01**
Education status (graduate)	0.72 (0.59–1.74)	P < 0.01**
Self-reported stroke^#^ (ref = no)	0.99 (0.57–1.20)	P = 0.99
Self-reported diabetes^#^ (ref = no)	0.85 (0.60–1.22)	P = 0.39
Physical activity^#^ (ref = active)	0.95 (0.77–1.16)	P = 0.59
General health status (ref = poor)	1.21 (0.90–1.34)	P = 0.04*
Visual problem^#^ (ref = yes)	0.98 (0.86–1.11)	P = 0.77
Hearing problem^#^ (ref = yes)	0.97 (0.85–1.10)	P = 0.65

Significant values are in [bold].

^#^Variable removed from the model, *P < 0.05, **P < 0.01.

## Discussion

The present study aimed to evaluate the test performance of the VST for the risk of future dementia development in comparison to established dementia screening tests, the HVLT and the SF-EMSE. The results of the study demonstrated that slower visual processing speeds, whether in simple or complex tasks, were linked to a higher risk of developing dementia in the future. While, the VSTs demonstrated low sensitivity and specificity in identifying those people who later at follow-up received a diagnosis of dementia in this cohort, the study also found that the HVLT and SF-EMSE were not highly sensitive or specific to identify future dementia risk.

It is important to acknowledge the limitations of this study. One such limitation concerns healthy volunteer bias and attrition which affect almost all such studies^28^. It is possible that individuals with (cognitive) disabilities may not have been willing or able to participate in the study, which may have resulted in a sample that is not representative of the general population^29^. Another limitation of the study is the overrepresentation of a single ethnicity (99.7% white) among the participants. This limited the ability to conduct ethnicity-stratified analyses and draw conclusions about the study population as a whole. It highlights the need for future research to be inclusive of a more ethnically diverse population. The use of self-reported outcomes, such as co-morbidity, physical activity, vision, and hearing, is another limitation of this study. These measures are known to be subject to inaccuracies and response bias, especially in people with dementia as they may have issues with recall. This may have affected the validity of the results and highlights the need for future research to consider the use of objective measures. On the other hand, our VST correlated with self-reported visual impairment suggesting validity of the self-report with regards to vision. However, the other limitations outlined above should be considered when interpreting the results. Future research should aim to address these limitations by including a more inclusive sample and using objective measures to enhance the validity of the results.

In the present analyses, the VSTs were found to have low sensitivity and specificity for identifying likely dementia. Possibly different data manipulations (e.g. removal of outliers, missing data and recoding variables) and inter-tester variability could account for low sensitivity of the VSTs in the present study. However, our earlier work in Oxford and Loughborough showed that VST performance was much worse in people with MCI and dementia, which was related to a loss of central field advantage speed of detection of stimuli^30^. Moreover, earlier the VST-complex was found to be a significant predictor of mortality in this cohort^31^. Of the 7144 individuals evaluated for complex visual sensitivity, the mortality risk of 5358 people scoring above the cutpoint was much lower than 1786 people with lower scores (7.3% and 13.8%, respectively). Dementia is the most common cause of death in the UK which could be related to this finding.

We found that lower visual processing speed was significantly associated with increased risk of future dementia in this cohort, although it did not show a better prediction of future dementia compared to the HVLT and SV-EMSE. Similar results were reported in previous analyses of this cohort by Hayat et al.^19^, using a different cut-off point for low performance associated with dementia risk (10th percentile). The ability of eight cognitive tests to identify the risk of future dementia was assessed in that study and it was reported that combining different cognitive tests including the VST could be a more appropriate method to identify future dementia risk.

Using logistic regression analyses, we found that complex VST was sensitive to several key risk factors for dementia, such as age, gender, education status, physical activity level, and self-reported visual problems. This might suggest that the test could be used with other screening tests in identifying the risk of future dementia and could in combination identify more risk and protective factors than each test used by itself. Physical activity and healthy ageing, defined as being free from a history of major chronic diseases and devoid of cognitive impairment, physical limitations, or mental health restrictions^32^, are associated with lower dementia risk, while stroke and diabetes (frequently found to be risk factors for dementia) were also associated with visual sensitivity^33,34^. It is not clear why gender and education were associated with visual sensitivity. Mechanisms could be that those with lifelong visual problems (without correction) were less able to obtain high levels of education^35^. Sex steroid loss after menopause could affect vision disproportionally in older women^36^. Studies have shown that hormone treatment including estrogen can benefit visual sensitivity^37,38^. However, long term use of combination sex hormones (oestrogens and progestagens for women with a womb) could result in increased dementia and breast cancer risk^39^.

All of the variables included in the regression models for the complex VST are associated with dementia risk and several are possible preventative factors (e.g. absence of stroke, diabetes, physical activity levels, short term hormone treatment (< 5–10 years, especially for women undergoing early menopause) and education^20,25^. However, some of these risk factors were not included in the models for EMSE and HVLT in the present cohort. The VST is also cross-culturally applicable, as is the HVLT^40^. The HVLT earlier had excellent sensitivity and specificity and was not affected by age, gender and education, unlike the MMSE^7^. In the current cohort these demographics independently of the dementia diagnoses did predict lower scores on both the VST and the HVLT. This suggests that adapted cut-offs for dementia screening (e.g. for an older age, females, those who received less education) would not be required for these tests. As suggested, inter-tester variability in how testing was conducted by various testers could be a further factor in explaining results with the dementia screening tests being less accurate than previously found, with sensitivity and specificity over 0.91, 0.98, respectively. In addition, these studies assessed people who had been diagnosed with dementia, whereas in the present study, risk for future dementia on average 9 years after cognitive assessment, was determined. Temporarily impaired cognition can be caused by other factors than dementia, such as infectious disease, which can reverse once these factors resolve or are treated explaining lower sensitivity overall.

In addition, issues in visual sensitivity are multifactorial, and are associated with ocular disorders including cataracts, glaucoma and age-related macular degeneration, but also with diabetes, hypertension and lipidemia affecting vascular perfusion of the eye and central processing, which morbidities are common in ageing^41,42^. In addition, loss of cholinergic function in AD could affect visual sensitivity as a co-morbid factor^43^. Cholinesterase inhibitors commonly used to treat dementia should then improve visual sensitivity^44^. Central factors in loss of visual sensitivity and perception, which might significantly affect activities of daily life and independent living, are described in more detail elsewhere^8^. These mechnisms and how they are related to dementia require more research.

Other studies of this and other cohorts including visual reaction time tests in dementia diagnostics found that combining the visual processing speed test with other neuropsychological tests is more effective way of indicating increased risk future dementia^19,45^. The Sydney Memory and Aging Study which is a volunteer cohort of 861 people without dementia at baseline followed up for 4 years investigated the ability of a task measuring visual RT to screen for the risk of future dementia in comparison with other screening tests, such as the Rey Auditory Verbal Learning Test (RAVLT), Logical Memory Delayed (LMD) recall, and the Trail Making Test (TMT)^46^. It was reported that higher simple visual RT (HR 1.53) and higher visual complex RT (HR 1.59) were both significantly associated with an increased risk of developing dementia within a 4-year period, as evidenced by the development of dementia in 48 cases at follow-up. These results also indicated that the accuracy of this approach in identifying people with or at risk of dementia, as measured by the area under the ROC curve, was comparable to the other more traditional neuropsychological measures, such as the RAVLT (AUC 0.72), TMT (AUC 0.64), and LMD (AUC 0.78). However, it should be noted that the AUC for the full neuropsychological battery was found to be superior (AUC 0.90). The prediction of functional decline by both RT measures combined was found to be equivalent to that of the full neuropsychological battery. Similarly, a recent study with a relatively smaller sample size (n = 123) was conducted to evaluate the effectiveness of multisensory RT in detecting MCI in comparison to the HVLT^47^. The results of the study were consistent with our findings. While the diagnostic power of sensorial RT (AUC 0.68, CI 95 0.53–0.84) was comparable to that of HVLT (AUC 0.73, CI 95% 0.34–0.93) in detecting cognitive impairment, the combination of the two tests resulted in improved sensitivity and specificity (AUC 0.82, CI 95% 0.66–0.97). This suggests that the combined use of multisensory RT and HVLT might present a more time-efficient and user-friendly alternative for individuals undergoing cognitive assessment. This combined approach might spare them from the laborious process associated with traditional neuropsychological testing, offering a more streamlined and accessible method for cognitive assessment. Also for both tests, tester training required is less than that for more extensive neuropsychological assessments requiring learned to interpretate clinical signs identified during this type of testing (e.g. how the test is performed and what the functional pattern of performance of different tests is in the individual cases).

Based on the results of the present study, visual sensitivity in dementia diagnostics and indicating risk in future has some promise, and could contribute to development of more effective tests that can identify the risk of future dementia. This may include further investigation into the use of the visual sensitivity test in combination with other neuropsychological tests, as well as the exploration of a comprehensive visual scanning test including visual sensitivity, contrast sensitivity and eye movements. Our earlier study showed that eye-tracking could be used as a dementia screening tool^48^. Additionally, it may be beneficial to investigate the sensitivity of VST in a large, multicentre cohort study including a range of ethnicities. Lastly, it is important to consider the possibility of multifactorial causes of visual sensitivity impairment and include these outcomes using objective assessment tools.

In conclusion, the present study aimed to evaluate the screening ability for future dementia risk using the VST compared to established dementia screening tests, the HVLT and the SF-EMSE. The results of the study revealed that the VST demonstrated lower sensitivity and specificity in identifying the risk of future dementia, but that the HVLT and SF-EMSE were also not highly sensitive or specific in screening for future dementia in this cohort. However, as the VST was significantly associated with more dementia risk factors, it could be integrated into the screening process for dementia risk and early diagnoses alongside other cognitive tests. Studies showed that incorporating VST in cognitive assessment can improve the sensitivity and specificity of the screening process, providing a more accurate assessment of cognitive impairment and identify risk perhaps more accurately to include protective factors to target for behaviour change. Furthermore, visual sensitivity impairment is a common occurrence in dementia and significant correlations have been found between visual sensitivity and dementia risk factors. Whether this test is sensitive to dementia treatments including lifestyle changes remains to be investigated in multi-ethnic cohorts.

### Supplementary Information

Supplementary Information.

## Data Availability

Data can be obtained upon a reasonable request. The authors are willing to provide the dataset through a Data Transfer Agreement to any legitimate researcher seeking access for replication analysis. Requests for data sharing or access should be formally submitted to the EPIC Management Committee via email at epic-norfolk@mrc-epid.cam.ac.uk.
