# Supplementary material for: Visual processing speed and its association with future dementia development in a population-based prospective cohort: EPIC-Norfolk
Source: Sci Rep. 2024 Feb 29;14:5016. doi: 10.1038/s41598-024-55637-x (PMC10904745; doi:10.1038/s41598-024-55637-x)
Supplement: Supplementary file 1 — Supplementary Information. [file 41598_2024_55637_MOESM1_ESM.docx]

**Appendix A: Table S1: Cox regressions for the association of cognitive measures and the risk of future dementia in the age-matched subgroup (aged ≥ 75 years)**

| **Cognitive tests, (n)**  (ref.=low score) | **Dementia (n)** | **Hazard ratio (95% CI)** | ***P* value** |
| --- | --- | --- | --- |
| VST-simple (1517)  VST-complex (1519)  HVLT (1826)  SF-EMSE (1996) | 225  226  288  313 | 1.36 (1.04, 1.77)  1.47 (1.12, 1.92)  2.84 (2.23, 3.61)  2.54 (2.01, 3.20) | **P<0.01****  **P<0.01****  **P<0.01****  **P<0.01**** |

Note: Ref: lower score of cut-off point was used as the reference group for comparison. All models adjusted for age, sex, and education.

**Appendix B: Receiver Operating Characteristics of cognitive tests**

**
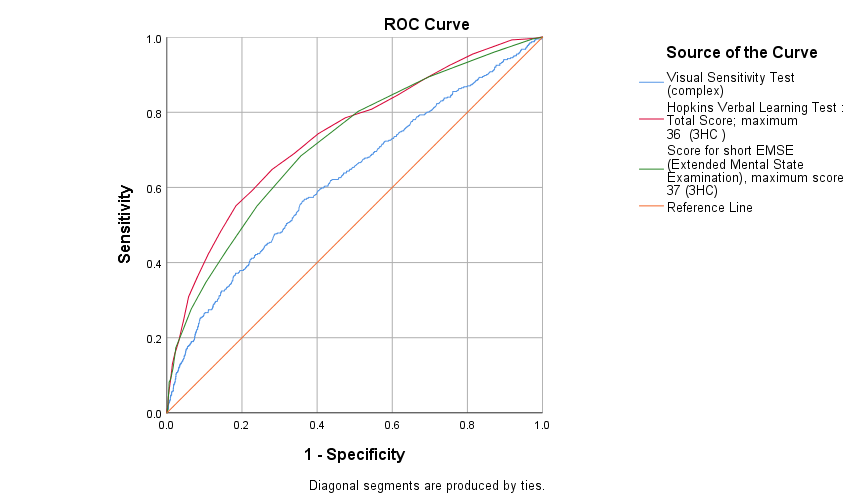
**

|  | Cut-off | Sensitivity | Specificity | AUC | 95% CI |
| --- | --- | --- | --- | --- | --- |
| VST-simple | 6.48 | 0.53 | 0.63 | 0.60 | (0.58-0.64) |
| VST-complex | 7.72 | 0.57 | 0.63 | 0.62 | (0.59-0.65) |
| HVLT | 20.5 | 0.65 | 0.72 | 0.74 | (0.72-0.77) |
| SF-EMSE | 32.5 | 0.68 | 0.64 | 0.72 | (0.69-0.75) |

**Appendix C: Spearman’s rank correlation matrix**


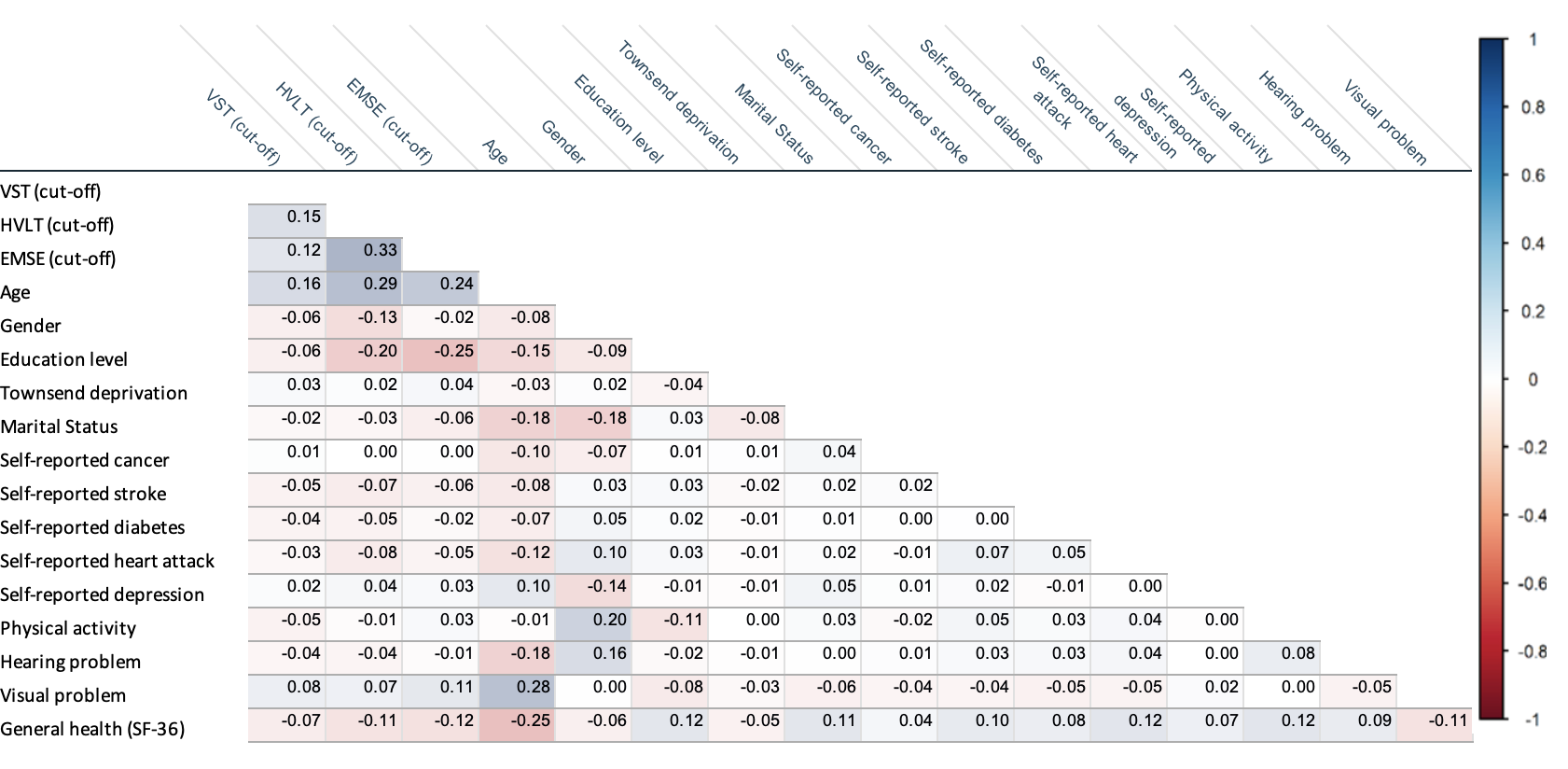


**Appendix D: Table S2: Logistic regression model for HVLT and SF-EMSE (CI = 95%)**

| **Variables** | ***P-value*** | **OR (95% CI)** |
| --- | --- | --- |
| **HVLT**  (R2=.18; χ2=11.50; P=0.18)  Age (years)  Gender (ref = men)  Marital status^#^ (ref = married)  Education status (ref.=No qualification)  Education status (O-A Level)  Education status (Graduate)  Self-reported heart attack^#^ (ref= no)  Self-reported stroke^#^ (ref= no)  Self-reported diabetes^#^ (ref= no)  Self-reported depression^#^ (ref= no)  General health status (ref = poor)  Visual problem^#^ (ref = yes)  Hearing problem (ref = yes)  **SF-EMSE**  (R2=.14; χ2=8.39; P=0.40)  Age (years)  Gender^#^ (ref = men)  Marital status^#^ (ref = married)  Education status (ref.=No qualification)  Education status (O-A Level)  Education status (Graduate)  Townsend DI  Self-reported stroke^#^ (ref= no)  Self-reported heart attack^#^ (ref= no)  Self-reported depression^#^ (ref= no)  Physical activity (ref = active)  General health status^#^ (ref = poor)  Visual problem (ref = yes)  Hearing problem^#^ (ref = yes) | **P<0.01****  **P<0.01****  P=0.93  **P<0.01****  **P<0.01****  **P<0.01****  P=0.76  P=0.38  P=0.24  P=0.29  P=0.07  P=0.25  **P<0.01****  **P<0.01****  P=0.21  P=0.72  **P<0.01****  **P<0.01****  **P<0.01****  **P<0.01****  P=0.09  P=0.63  P=0.91  **P<0.01****  P=0.20  **P<0.01****  P=0.96 | 1.09 (1.08-1.10)  0.50 (0.42-0.58)  1.01 (0.83-1.22)  -  0.52 (0.44-0.58)  0.21 (0.16-0.28)  1.07 (0.68-1.67)  0.75 (0.40-1.42)  0.78 (0.51-1.19)  0.89 (0.71-1.11)  0.82 (0.66-1.02)  0.90 (0.77-1.07)  1.30 (1.10-1.54)  1.06 (1.05-1.07)  0.92 (0.81-1.05)  1.03 (0.89-1.19)  -  0.50 (0.44-0.58)  0.22 (0.18-2.67)  1.04 (1.01-1.07)  0.61 (0.35-1.77)  0.90 (0.62-1.34)  1.01 (0.85-1.20)  1.01 (1.00-1.01)  1.12 (0.94-1.34)  1.20 (1.06-1.38)  1.00 (0.88-1.14) |

#: Variable removed from the model, *: P<0.05, **: P<0.01
